# Does early orchidopexy reduce risk of testicular cancer?

**Published:** 2008

**Authors:** John S. Banerji, J. Chandra Singh

**Affiliations:** Department of Urology, Christian Medical College, Vellore, India

## SUMMARY

Petterson *et al*.,[1] identified 16,983 Swedish men from the Swedish Hospital Discharge Register who underwent orchidopexy for undescended testis, between 1964 to 1999. As almost all patients in Sweden are treated at public hospitals, this data was fairly comprehensive. Subjects who underwent orchidopexy before the age of 20 years were included. Two hundred and five men who emigrated from Sweden after surgery but did not immigrate back before the start of their follow-up and one who had testicular cancer at the time of beginning the study were excluded. Other exclusion includes those with ambiguity on information on date of orchiodopexy or sex. The subjects were followed from 15 years of age or the age of orchidopexy plus one year, whichever occurred earlier, till the date of diagnosis of testicular cancer, upper age limit of 55 years, emigration, death, or December 2000. The authors have calculated the expected period-specific incidence for five-year age periods, based on the incidence in the Swedish general population. Based on the observed number of cases in the study population, the relative risk was estimated. They used a number of covariate analyses, including separation of the follow-up into three distinct calendar periods, time of orchidopexy and the place where orchidopexy was performed. Further statistical analysis using Schoenfeld residuals indicated that the assumption of hazard ratio was correct, and all such confounders were eliminated.

The mean follow-up was 12.4 years. Mean age at orchidopexy was 8.6 years. Though only 20 cases was expected in the study group based on the incidence in the general population, 56 were documented. The standardized incidence ratio of testicular cancer in those undergoing orchidopexy before the age of 13 years was 2.23, as opposed to those above the age of 13 years, where it was almost double at 5.40.

## COMMENTS

This population-based study from Sweden is one of the largest epidemiological studies. As healthcare is mostly through public hospitals in Sweden, it was possible to have such a large number. Analysing, using smaller age intervals, there was an increased risk compared to the general population. Histological studies demonstrate germ cell damage, thus favoring orchidopexy to be carried out before the age of two years and now probably even by six months. But there was a dearth of data to support the impact of the timing of orchidopexy on the risk of malignancy. This cohort study undertook the daunting task of answering that question. In the 0-6 years’ age group, the number of cancers is too small to comment on. In a study including 794 men and nine regions in England and Wales, the United Kingdom Testicular Cancer Study Group concluded that there was indeed an elimination of risk in men who had an orchidopexy before the age of 10 years.[2] Walsh *et al*., in a meta-analysis prior to the publication of this study concluded that the risk of cancer increases nearly six times if orchidopexy was delayed until after age 10 to 11 years or was never performed.[3] Swerdlow *et al*., in a study from the Hospital for Sick Children, concluded that though the relative risk of testicular cancer fell significantly 15 years after orchidopexy, there was no decrease in relative risk with younger age at orchidopexy.[4] But Myrup *et al*., observed that the risk of testicular cancer did not increase with age at orchidopexy,[5] in a study design that was similar to that of Pettersson *et al*.,[1] with 21,488 subjects from Denmark. Though it is largely believed that the risk of germ cell cancer is determined in utero, the results of this study put forward a hypothesis that puberty possibly brings forth another crucial event in testicular carcinogenesis. Till the final word is said, it would be prudent to perform orchidopexy at the earliest possible opportunity, when it is decided that the possibility of spontaneous descent is remote.
